# Clinical effectiveness of sodium bicarbonate therapy on mortality for septic patients with acute moderate lactic acidosis

**DOI:** 10.3389/fphar.2022.1059285

**Published:** 2023-01-09

**Authors:** Sai Huang, Bo Yang, Yaojun Peng, Qinrui Xing, Lili Wang, Jing Wang, Xuan Zhou, Yuan Yao, Li Chen, Cong Feng

**Affiliations:** ^1^ National Clinical Research Center of Geriatric Diseases, Chinese PLA General Hospital, Beijing, China; ^2^ Department of Hematology, Fifth Medical Center of Chinese PLA General Hospital, Beijing, China; ^3^ Department of Thoracic Surgery, First Medical Center, General Hospital of PLA, Beijing, China; ^4^ Department of Emergency, First Medical Center of Chinese PLA General Hospital, Beijing, China; ^5^ Department of Emergency, Hainan Hospital of Chinese PLA General Hospital, Sanya, China; ^6^ Department of General Medicine, First Medical Center of General Hospital of People’s Liberation Army, Beijing, China; ^7^ Hospital Management Institute, Medical innovation research department, Chinese PLA General Hospital, Beijing, China; ^8^ Department of General Medicine, Hainan Hospital of General Hospital of People’s Liberation Army, Beijing, China; ^9^ State Key Laboratory of Kidney Diseases, National Clinical Research Center of Kidney Diseases, General Hospital of People’s Liberation Army, Beijing, China

**Keywords:** acute moderate lactic acidosis, mortality, sodium bicarbonate administration, sepsis, septic shock

## Abstract

**Objective:** This study aimed to explore the effectiveness of sodium bicarbonate (SB) administration on mortality in septic patients with acute moderate lactic acidosis (MLA).

**Methods:** The large ICU database (MIMIC-IV) was retrospectively analyzed. Patients with sepsis and acute MLA were identified. Propensity score analysis (PSA) was performed to explain baseline differences in the probability of accepting or not accepting SB. The edge structured cox model (MSCM) was used to adjust for baseline and time-varying confounding variables. The primary outcome was the ICU and hospital mortality. The subgroup of septic shock was also investigated.

**Results:** A total of 512 septic patients with acute MLA were identified in this study, including 160 in the SB group and 352 in the non-SB group. In the PSA, SB administration was associated with reduced ICU (HR .58, 95% CI 0.38–.89; *p* <.05) and hospital (HR .67, 95% CI 0.47–.95; *p* <.05) mortality in septic patients with acute MLA. In the subgroup, the results were similar with septic patients. In the MSCM, SB administration could also improve the ICU (HR .35, 95% CI 0.16–.75; *p* <.01) and (HR .50, 95% CI 0.28–.88; *p* <.05) mortality on septic patients. However, In the subgroup, SB administration could only be found associated with improved hospital (HR .44, 95% CI 0.20–.97; *p* <.05) survival in septic shock.

**Conclusion:** SB administration treatment could reduce ICU and hospital mortality of septic patients with acute MLA. Meanwhile, it could also improve hospital survival in the subgroup of septic shock patients with acute MLA.

## Introduction

Sepsis are major health care problems in the intensive care unit (ICU), affecting millions of people worldwide every year, of which one-third to one-sixth die (Fleischmann et al., 2016; Rhee et al., 2017; Fleischmann-Struzek et al., 2020). Lactic acidosis, as one of the most common types of moderate lactic acidosis, was also commonly observed in sepsis with a strikingly high mortality according to the different definitions (Luft et al., 1983; Fall and Szerlip, 2005). Early and appropriate treatment after sepsis and lactic acidosis could improve the prognosis.

Sodium bicarbonate solution (SB) is commonly applied in fluid resuscitation and correcing the disorders of acid and base in the early stage after sepsis. While SB therapy was widely investigated for its relationship with mortality in severe septic patients (Ganesh et al., 2016; Jaber et al., 2018; Zhang et al., 2018; Fujii et al., 2021; Yagi and Fujii, 2021), sepsis with lactic acidosis has been less well investigated. Only two small RCTs have compared the effects of sodium bicarbonate and equimolar saline in treating lactic acidosis and no difference was found in hemodynamic indicators or vasopressin consumption (Cooper et al., 1990; Mathieu et al., 1991). In the update of the Surviving Sepsis Campaign guidelines 2021, Evans et al. (2021) suggest against using SB treatment to make improvement in haemodynamics or to decrease the consumption of vasopressor for adults with septic shock and lactic acidemia induced by hypoperfusion (INVALID CITATION, 2018). However, the effect of SB therapy on death rate when used in most septic patients with moderate lactic acidosis (MLA) is still not known.

According to the aforementioned information, the purpose of our study was exploring the effects of SB administration on the death rate of the septic patients who had acute MLA and in the subgroup of septic shock. Since SB treatment is a time-weighted predictive factor which depends on bicarbonate concentration (BC), lactate, PaCO_2_ and pH in blood, the SB administration records, time-dependent covariates, and baseline data were explained by using the marginal structural cox model (MSCM) (Robins et al., 2000; de Keyser et al., 2014; Karim et al., 2014).

## Materials and methods

### The design of the research and database

A retrospective design and MIMIC-IV, v0.4 database were used., This research included 76540 critically ill patients from six ICUs in a single center institution (Beth Israel Deaconess Medical Center, bidmc; Boston, MA) between 2008 and 2019 (Johnson et al., 2016; Johnson et al., 2018; Johnson et al., 2020). Since the database we analyzed here has been approved for public use by the institutional review board (IRB), there is no need to additionally provide IRB approval.

### Study cohort

All admissions in the MIMIC-IV database were included. Non-etheless, only the admissions to ICU recorded for the first time were used for the analysis (Zhang et al., 2018). Inclusion criteria: 1) sepsis was diagnosed on the admission to ICU; 2) onset of acute MMA (within a few days (Yagi and Fujii, 2021) with 7.2 ≤ pH < 7.3 and BC <20 mmol/L; 3) with lactate >2.2 mmol/L; 4) no respiratory acidosis (PaCO_2_ < 50 mmHg) (Zhang et al., 2018); 5) without stage 2 or 3 AKI (the diagnosis and staging of AKI was according to the level of serum creatinine following the Kidney Disease: Improving Global Outcomes) (Mathieu et al., 1991). Patients’ pH, BC, lactate, serum creatinine and PaCO_2_ were documented after admission to ICU within 48 h (Singer et al., 2016). The sepsis was defined as a condition with “life-threatening organ dysfunction caused by a dysregulated host response to infection” (Singer et al., 2016). If the results of various examination were documented, only the highest level of lactate and PaCO_2_, and the lowest level of pH and BC were utilized. We excluded the patients with cardiac arrest or ICU stay >100 days (Zhang et al., 2018).

### Variables

The variables chosen from MIMIC-IV database were the data documented within 24 h after the patient was admitted to the ICU, including sex, body mass index (BMI), age, comorbidity after admission to ICU, mechanical ventilation, and vasopressor consumption. Acute kidney injury (AKI) was defined according to the Kidney Disease: Improving Global Outcomes (Kellum and Lameire, 2013). The shock index (shock index >1.0) was used to evaluate the degree of shock on the first day of admission to ICU (Allgöwer and Burri, 1946). Lactate solution treatment in the first 24 h was also taken into account. In the establishment of the mixed-effects model, the admission year was used as a random factor (SerpaNeto et al., 2018).

Laboratory indicators including pH, lactate, PaCO_2_, and BC were documented during the whole period of ICU stay. The result and examination time of the lab tests were extracted from the MIMIC-IV. If the test was conducted for more than one time, the highest PaCO_2_ and lactate values, and the lowest pH and BC values every day were used in the analysis.

### Outcome measures

The primary outcome measure was the mortality of the patients during hospital stay. The survival rate when patients were discharged from the hospital were also an outcome measure.

### Statistical analysis

The cohorts were assigned into intervention (SB) and control (non-SB) group based on SB treatment during the first 48 h after the admission to ICU. Continuous variables were presented as median value with interquartile range (IQR) or mean value with standard deviation. Mann-Whitney *U* test or Student’s t-test was utilized to make comparison between groups. Categorical data were presented as relative frequency (%) and absolute frequency (n). Fisher’s exact test or Chi-square test was employed to analyze the data.

The baseline differences in the possibility of applying SB treatment were explained by using the propensity score analysis (PSA) (Zhang, 2017). In PSA, the patients in SB group were given SB during the first 48 h of ICU stay. The data in PSA are presented in Table 1. The propensity scores of the two groups were matched by using the nearest neighbor method. The parameters (*p* < .05) were used to adjust the residual imbalance in Cox regression model. Clinical professionals were consulted to determine the possible confounders.

**TABLE 1:** Baseline differences between two groups before propensity score matching.

Key characteristics	Non-SB group (n)	SB group (n)	*p*-value
Demographic information			
Final cohort (n)	352	160	—
Age, years (median, (IQR))	67.50 (23.25)	68.00 (22.25)	.57
Male gender (n (%))	189 (53.69)	81 (50.63)	.58
BMI, (median, (IQR))	28.39 (9.69)	28.86 (8.21)	.53
Admission period, n (%)			.15
Before 2014	217 (61.65)	236 (65.37)	—
2014–2019	135 (38.35)	125 (34.63)	—
Comorbidities (n (%))			
Hypertension	160 (45.45)	54 (33.75)	<.05
Diabetes	123 (34.94)	53 (33.13)	.76
Congestive heart failure	103 (29.26)	48 (30.00)	.95
Chronic pulmonary disease	50 (14.20)	23 (14.38)	1.00
Chronic kidney disease	76 (21.59)	38 (23.75)	.67
Chronic liver disease	24 (6.82)	14 (8.75)	.55
The incidence of shock status, (n (%))			
Shock	242 (68.75)	124 (77.50)	.05
Additional respiratory and hemodynamic support, (n (%))			
Mechanical ventilation	309 (87.78)	138 (86.25)	.73
Vasopressors	307 (87.22)	139 (86.88)	1.00
Laboratory values, (median, (IQR))			
Minimum PaO2, %	79.00 (28.25)	75.00 (25.25)	<.05
Maximum PaCO2, %	44.00 (11.00)	43.00 (12.25)	.85
Minimum pH	7.24 (.07)	7.21 (.11)	<.01
Minimum bicarbonate concentration, mmol/L	16.00 (4.00)	14.00 (5.00)	<.01
Maximum lactate, mmol/L	4.00 (2.53)	5.35 (5.05)	<.01
Lactate solution, (n (%))	175 (49.72)	56 (35.00)	<.01

BMI: body mass index.

SB therapy during ICU stay was a time-weighted variable in MSCM. Sex, BMI, age, comorbidities, mechanical ventilation application and vasopressor were possible confounders at baseline. These data were documented during the first 24 h of ICU stay. BC, pH, lactate, and PaCO_2_ tested during ICU stay varied with time increased. The selection and confounding bias resulted from informative censoring were corrected by using “ipw” package (version 1.0-11) and Inverse probability weighting estimation in the R (version 4.0.0) (Robins et al., 2000). The variables used in MSCM were list in the Supplementary Tables S8–S10.

There were missing data on (<25%) pH, PaO2, BMI, PaCO2 and shock index (Supplementary Table S1). Various imputation procedures were used for these missing variables. Multiple imputation was carried out using the prediction mean matching method of continuous variables and the logistic regression method of categorical data, and 5 databases were established. After multiple imputation, the multivariate model was replicated in 5 databases and the pooled results were obtained (SerpaNeto et al., 2018).

MSCM and PSA analyses were used to conduct subgroup analysis by grouping patients into septic shock patients with acute moderate lactic acidosis.

The statistical analyses were all conducted by applying R package (version 4.0.0). *p* < .05 was regarded as statistically significant different.

## Results

### Study cohort

**FIGURE 1:**
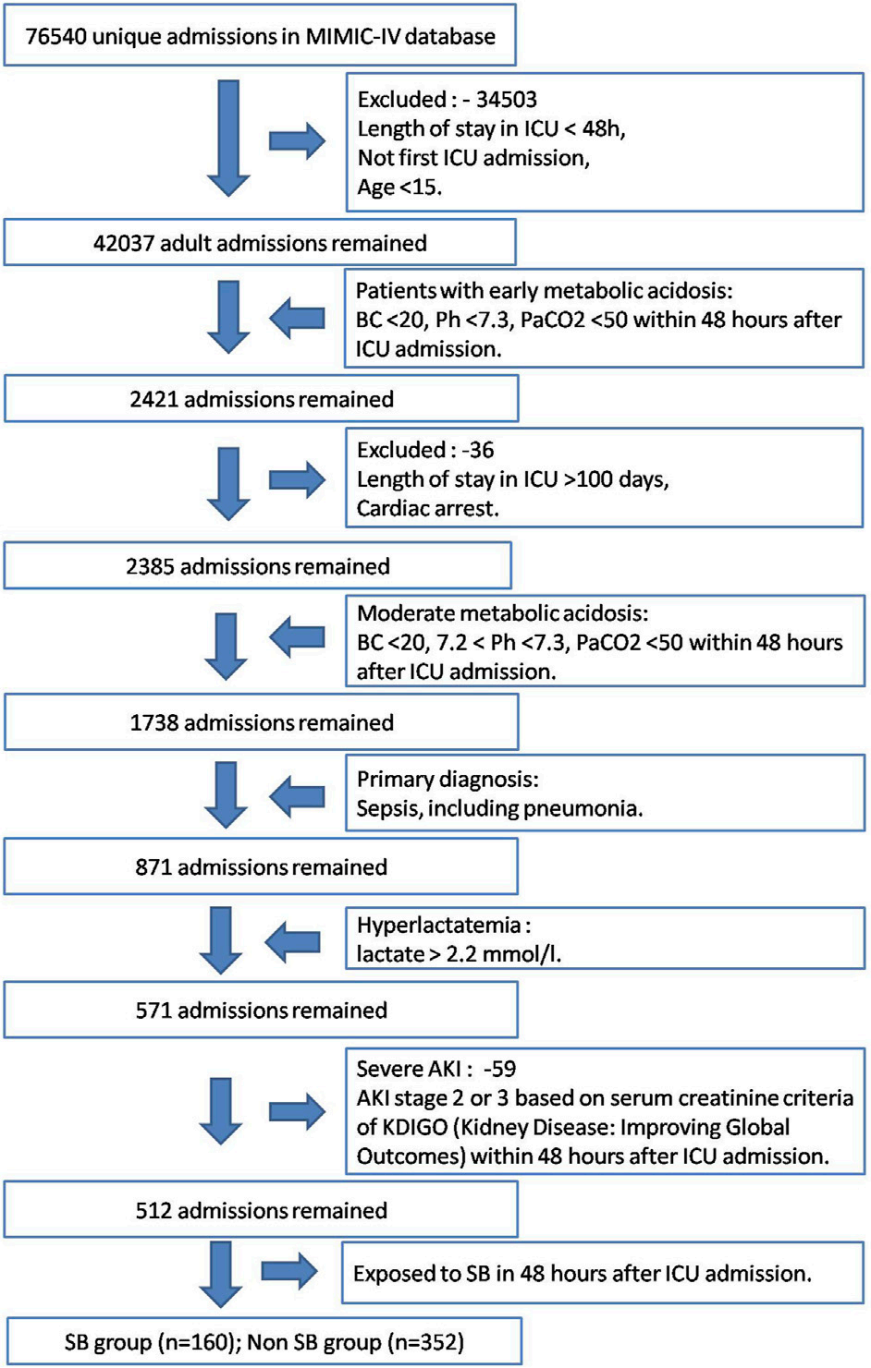
Study cohort selection workflow of MIMIC-IV database based on the designed inclusion and exclusion criteria.

A total of 512 septic patients from the MIMIC-IV were selected who had acute moderate lactic acidosis during the first 48 h of ICU stay. Among them, 160 patients underwent SB therapy during the first 48 h, and 352 patients didn’t underwent the therapy (Figure 1).

Table 1 presented the information in the two groups at baseline. The hypertension percentage was lower in SB group [54 (33.75%) vs. 160 (45.45%); *p* <.05] in comparison with non-SB group. In the first 24 h of ICU stay, the incidence of shock was higher in SB group [124 (77.50%) vs. 242 (68.75%); *p* = .05] compared with non-SB group. There was no statistically significant difference in mechanical ventilation application or vasopressors between SB and non-SB groups. The minimum pH [7.21 (.11) vs. 7.24 (.07), *p* <.01], PaO2 [75.00 (25.25) vs. 79.00 (28.25), *p* <.01] and BC [14.00 (5.00) vs. 16.00 (4.00), *p* <.01] were remarkably reduced, and lactate level was remarkably increased [5.35 (5.05) vs. 4.00 (2.53), *p* <.01] in SB group compared with the non-SB group. The application of lactate solution was markedly increased in the non-SB group [56 (35.00) vs. 175 (49.72); *p* <.01] in comparison with SB group. There was no statistically significant difference in admission period between the two groups. There was no participant having multiple times of admission in the cohort.

### Propensity score analysis

In PSA, 160 patients from SB and 160 patients from non-SB groups were matched, and the imbalance between the two groups was largely improved (Supplementary Table S2). Cox proportional hazard model was applied due to the remaining imbalances. It was demonstrated that SB administration was highly related with decreased mortality of the septic patients with acute MLA in ICU (HR: .58; 95% CI: .38–.89; *p* <.05) and in hospital (HR: .67; 95% CI: .47–.95; *p* <.05) (Table 2; Table 3). In the subgroup of septic shock patients who had acute MLA, it was also presented that SB administration was in a close relationship with increased survival rate in ICU stay (HR: .53; 95% CI: .32–.89; *p* <.05) and hospital stay (HR: .58; 95% CI: .38–.89; *p* <.05) survival (Table 2; Table 3).

**TABLE 2:** Association of early sodium bicarbonate infusion and ICU mortality in the overall and subgroup by using propensity score analysis.

Overall and subgroup	Hazard ratio	Lower.95	Upper.95	p-value
Sepsis with acute moderate lactic acidosis (n = 360)	.58	.38	.89	<.05
Septic shock with acute moderate lactic acidosis (n = 248)	.53	.32	.89	<.05

**TABLE 3:** Association of early SB infusion and hospital mortality in the overall and subgroups by using propensity score analysis.

Overall and subgroups	Hazard ratio	Lower.95	Upper.95	*p*-value
Sepsis with acute moderate lactic acidosis (n = 360)	.67	.47	.95	<.05
Septic shock with acute moderate lactic acidosis (n = 248)	.58	.38	.89	<.05

### Results of marginal structural cox model

**FIGURE 2:**
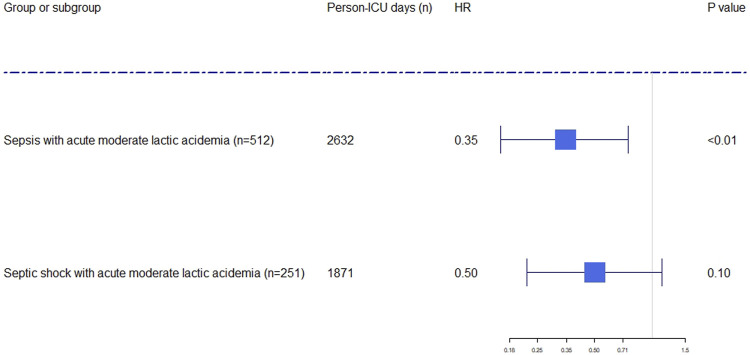
Forest plot showing the effect of sodium bicarbonate administration on ICU mortality in septic and septic shock patients with acute moderate lactic acidosis. The hazard ratios were estimated using the marginal structural cox model. Person-days were the days of ICU and hospital length of stay. The x-axis tick marks follow a logarithmic scale. HR: hazard ratio.

**FIGURE 3:**
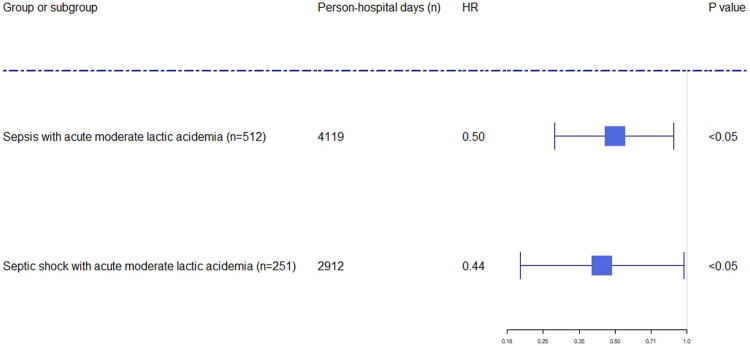
Forest plot showing the effect of sodium bicarbonate administration on hospital mortality in septic and septic shock patients with acute moderate lactic acidosis. The hazard ratios were estimated using the marginal structural cox model. Person-days were the days of ICU and hospital length of stay. The x-axis tick marks follow a logarithmic scale. HR: hazard ratio.

SB administration and time-dependent confounding factors were analyzed by using MSCM. The results of MSCM demonstrated the relationships between SB administration and decreased mortality in ICU (HR: .35; 95% CI: .16–.75; *p* <.01) and hospital (HR: .50; 95% CI: .28–.88; *p* <.05) in the septic patients who had acute MLA (Figures 2, 3, Supplementary Table S3). In the subgroup (Figures 2, 3, Supplementary Table S4), significant association was only found between SB administration and reduced hospital mortality (HR: .44; 95% CI: .20–.97; *p* <.05), but not found with ICU mortality (HR: .50; 95% CI: .22–1.13; *p* = .10). The key factors of SB administration for predicting hospital and ICU mortality in septic patients with acute MLA, and for the mortality in hospital in septic patients who had acute MLA were showed in Supplementary Tables S5–S7. Meanwhile, the predictors of the use of sodium bicarbonate infusion at each time point during hospital and ICU stay in septic patients with acute MLA and during hospital stay in septic shock patients with acute MLA was showed in Supplementary Tables S8–S10.

## Discussion

According to our results, it was found that there was strong relationship between SB administration and decreased mortality during ICU and hospital stay in adult septic patients who had acute MLA. SB administration was also associated with reduced hospital mortality in septic shock patients who had acute MLA in the subgroup. Our research presented that lactic acidosis was commonly found in the patients at critically ill status who suffered from septic shock and sepsis, and SB administration was often employed in these patients, however, there was no sufficient evidence of the effect on mortality (Forsythe and Schmidt, 2000; Boyd and Walley, 2008; Evans et al., 2021). Hence, our study was the first proving that SB administration could improve the survival of septic patients who had acute MLA in both ICU and hospital, and also could reduce hospital mortality for septic shock patients who had acute MLA.

SB administration was extensively used in clinical practice for the treatment of severe moderate lactic acidosis in ICU(9). Nevertheless, no sufficient evidence was found to prove its effects on mortality in septic patients who had MLA. Cooper et al. (1990) presented that sodium bicarbonate therapy could elevate PCO2 and pH without benefits in cardiac output or blood pressure. Mathieu et al. (1991) could not found any change in hemodynamic parameters. In a retrospective observational study, Zhongheng Zhang et al. (2018) also did not find the significant improved effect on mortality rate for sepsis patients who had severe lactic acidosis. Current research showed that SB administration was not helpful to the sepsis patients who had acute MLA but was also related to improved clinical outcomes for septic shock patients. It was demonstrated by our study that SB therapy was advantageous for septic shock and sepsis patients who had acute MLA.

Based on the latest review, Kosuke Yagi and Tomoko Fujii concluded that the presence of moderate lactic acidosis was common in ICU, and the mortality was even higher in moderate lactic acidosis rather than severe sepsis (Yagi and Fujii, 2021). However, high quality evidence of SB administration for moderate lactic acidosis are still lacking. The standard practice of SB could be effective in both and moderate lactic acidosis. Our observations provided supportive proof that SB administration could be applied in sepsis patients who had acute MLA, especially in the patients suffering from septic shock.

There were some additional strengths in our study. Firstly, the data were extracted from the MIMIC-IV database which ensured high quality of the and a large cohort of patients. The possible relationship between SB therapy and mortality could be observed. Secondly, MSCM and PSA were used in this research to explain the data at baseline and the time-dependent confounders. Thirdly, the subgroup analysis was conducted and potent evidence was found in septic shock patients with acute MLA.

There were also limitations in this study. Firstly, the cohort in this study is only from one institution. Randomized controlled clinical trials could provide more persuasive evidence. Secondly, some data (liquid input and output, etc.) were missing due to the unavailability of data in the database. Thirdly, PSA and MSCM were applied to balance key confounding variables, however, residual confounding variables could not be completely avoided since the observational study design was adopted in this study. Fourth, the data collection time in MIMIC-IV was more than 10 years, during which there was a huge change in clinical care. Fifth, missing data was also a limitation. There was no difference in baseline data between the combined estimated and source datasets. Sixth, due to incorrect and missing data, sofa scores were not included in the evaluation variables. Other complete and correct variables, such as comorbidity, additional hemodynamic and respiratory support, which were closely related to the SOFA score evaluation, were used in the analysis. Seventh, the effect of SB treatment which as a time-weighted predictive factor was only observed focusing on the initial 48 h in this study, but not included other time windows that might also impact on mortality. Eighth, Multiple imputation can be quite different when it is applied to cross-sectional data or longitudinal data. Last, the findings of this research need to be further confirmed by rigorous clinical studies, such as randomized clinical trials.

## Conclusion

SB therapy could decrease the mortality rate in hospital and ICU stay in septic patients who had acute MLA. Meanwhile, it could also improve hospital survival in the subgroup of septic shock patients with acute MLA. Randomized controlled clinical trials are further required to validate our findings.

## Data Availability

The original contributions presented in the study are included in the article/Supplementary Material, further inquiries can be directed to the corresponding authors.
