# Supplementary material for: Clinical effectiveness of sodium bicarbonate therapy on mortality for septic patients with acute moderate lactic acidosis
Source: Front Pharmacol. 2023 Jan 9;13:1059285. doi: 10.3389/fphar.2022.1059285 (PMC9868412; doi:10.3389/fphar.2022.1059285)
Supplement: Supplementary file 1 [file Table1.DOC]

**Supplemental Tables of Contents**

Supplemental Table 1: Information about data missingness.

Supplemental Table 2: Baseline differences between two groups after propensity score matching.

Supplemental Table 3: Results of marginal structural cox model for ICU and hospital mortality in septic patients with acute moderate lactic acidosis..

Supplemental Table 4: Results of marginal structural cox model for ICU and hospital mortality in patients with septic shock and acute moderate metabolic acidosis.

Supplemental Table 5: The important variables of SB administration for based on marginal structural cox model for predicting ICU mortality in septic patients with acute moderate lacitc acidosis.

Supplemental Table 6: The important variables of SB administration for based on marginal structural cox model for predicting hospital mortality in septic patients with acute moderate lacitc acidosis.

Supplemental Table 7: The important variables of SB administration for based on marginal structural cox model for predicting hospital mortality in septic shock patients with acute moderate lacitc acidosis.

Supplemental Table 8: Predictors of the use of sodium bicarbonate infusion at each time point during ICU stay in septic patients with acute moderate lactic acidosis.

Supplemental Table 9: Predictors of the use of sodium bicarbonate infusion at each time point during hospital stay in the septic patients with acute moderate lactic acidosis

Supplemental Table 10: Predictors of the use of sodium bicarbonate infusion at each time point during hospital stay in the septic shock patients with acute moderate lactic acidosis

.

**Supplemental Table 1. Information about data missingness.**

| **Variables** | **Missing percentage, n(%)** |
| --- | --- |
| Cohort (n) | 512 |
| Age | 0 (0%) |
| Gender | 0 (0%) |
| BMI | 11 (2.15%) |
| Admission period | 0 (0%) |
| Hypertension | 0 (0%) |
| Diabetes | 0 (0%) |
| Congestive heart failure | 0 (0%) |
| Chronic pulmonary disease | 0 (0%) |
| Chronic kidney disease | 0 (0%) |
| Chronic liver disease | 0 (0%) |
| AKI | 0 (0%) |
| Renal replacement therapy | 0 (0%) |
| Shock index | 28 (5.47%) |
| Mechanical ventilation | 0 (0%) |
| Vasopressors | 0 (0%) |
| Minimum PO2 | 19 (3.71%) |
| Maximum PCO2 | 19 (3.71%) |
| Minimum pH | 1 (0.20%) |
| Minimum bicarbonate concentration | 0 (0%) |
| Maximum lactate | 0 (0%) |
| Lactate solution | 0 (0%) |
| Use of sodium bicarbonate | 0 (0%) |
| Length of stay in ICU | 0 (0%) |
| Length of stay in hospital | 0 (0%) |
| ICU mortality | 0 (0%) |
| Hospital mortality | 0 (0%) |

BMI: body mass index; AKI: acute kidney injury

**Supplemental Table 2.** Baseline differences between two groups after propensity score matching.

| **Key Characteristics** | **Non-SB group (n=)** | **SB group (n=)** | **P-value** |
| --- | --- | --- | --- |
| Demographic information: |  |  |  |
| Final cohort (n) | 160 | 160 | - |
| Age, years (median, (IQR)) | 67.00 (24.25) | 68.00 (22.25) | 0.81 |
| Male gender (n(%)) | 180 (49.86) | 81 (50.63) | 0.50 |
| BMI, (median, (IQR)) | 28.08 (9.37) | 28.86 (8.21) | 0.56 |
| Admission period, n(%) |  |  | 0.26 |
| Before 2014 | 120 (75.00) | 236 (65.37) | - |
| 2014-2019 | 40 (25.00) | 125 (34.63) | - |
| Comorbidities (n(%)): |  |  |  |
| Hypertension | 61 (38.13) | 54 (33.75) | 0.48 |
| Diabetes | 54 (33.75) | 53 (33.13) | 1.00 |
| Congestive heart failure | 48 (30.00) | 48 (30.00) | 1.00 |
| Chronic pulmonary disease | 19 (11.88) | 23 (14.38) | 0.62 |
| Chronic kidney disease | 35 (21.88) | 38 (23.75) | 0.79 |
| Chronic liver disease | 12 (7.50) | 14 (8.75) | 0.84 |
| The incidence of shock status, (n(%)): | | | |
| Shock | 123 (76.88) | 124 (77.50) | 1.00 |
| Additional respiratory and hemodynamic support, (n(%)): | | | |
| Mechanical ventilation | 140 (87.50) | 138 (86.25) | 0.87 |
| Vasopressors | 133 (83.13) | 139 (86.88) | 0.43 |
| Laboratory values, (median, (IQR)): | | | |
| Minimum PaO2, % | 75.00 (26.25) | 75.00 (25.25) | 0.50 |
| Maximum PaCO2, % | 43.00 (11.00) | 43.00 (12.25) | 0.78 |
| Minimum pH | 7.21 (0.07) | 7.21 (0.11) | <0.05 |
| Minimum bicarbonate concentration, mmol/L | 15.00 (2.73) | 14.00 (5.00) | <0.05 |
| Maximum lactate, mmol/L | 4.05 (3.20) | 5.35 (5.05) | <0.01 |
| Lactate solution, (n(%)) | 59 (36.88) | 56 (35.00) | 0.82 |

BMI: body mass index

**Supplemental Table 3. Results of marginal structural cox model for ICU and hospital mortality in septic patients with acute moderate lactic** acidosis.

| Outcomes | Number of patients | Number of person-days | Hazard ratio | Lower.95 | Upper.95 | P value |
| --- | --- | --- | --- | --- | --- | --- |
| ICU mortality | 512 | 2632 | 0.35 | 0.16 | 0.75 | <0.01 |
| Hospital mortality | 512 | 4119 | 0.50 | 0.28 | 0.88 | <0.05 |

:

**Supplemental Table 4. Results of marginal structural cox model for ICU and hospital mortality in patients with septic shock** and acute moderate metabolic acidosis.

| Outcomes | Number of patients | Number of person-days | Hazard ratio | Lower.95 | Upper.95 | P value |
| --- | --- | --- | --- | --- | --- | --- |
| ICU mortality | 251 | 1871 | 0.50 | 0.22 | 1.13 | 0.10 |
| Hospital mortality | 251 | 2912 | 0.44 | 0.20 | 0.97 | <0.05 |

**Supplemental Table 5. The important variables of SB administration for based on marginal structural cox model for predicting ICU mortality in septic patients with acute moderate lacitc acidosis.**

| Variables | Hazard ratio | Lower.95 | Upper.95 | P value |
| --- | --- | --- | --- | --- |
| Use of sodium bicarbonate | 0.35 | 0.16 | 0.75 | <0.01 |
| Use of vasopressors | 2.08 | 1.16 | 3.76 | <0.05 |
| Age with 10 years increase | 1.16 | 1.02 | 1.33 | <0.05 |
| PCO2 (mmHg) on admission | 1.04 | 1.01 | 1.06 | <0.01 |
| Lactate (mmol/L) on admission | 0.85 | 0.80 | 0.91 | <0.01 |
| Lactate (mmol/L) with 0.1 increase | 1.03 | 1.03 | 1.04 | <0.01 |

**Supplemental Table 6. The important variables of SB administration for based on marginal structural cox model for predicting hospital mortality in septic patients with acute moderate lacitc acidosis.**

| **Variables** | Hazard ratio | Lower.95 | Upper.95 | P value |
| --- | --- | --- | --- | --- |
| Use of sodium bicarbonate | 0.50 | 0.28 | 0.88 | <0.05 |
| Diabetes | 1.45 | 1.04 | 2.02 | <0.05 |
| Hypertension | 0.68 | 0.47 | 0.99 | <0.05 |
| Use of vasopressors | 1.56 | 1.02 | 2.39 | <0.05 |
| Age with 10 years increase | 1.26 | 1.12 | 1.42 | <0.01 |
| PCO2 (mmHg) on admission | 1.03 | 1.01 | 1.05 | <0.01 |
| Lactate (mmol/L) on admission | 0.85 | 0.81 | 0.90 | <0.01 |
| Lactate solution (L) with 1 increase | 0.88 | 0.80 | 0.96 | <0.01 |
| Lactate (mmol/L) with 0.1 increase | 1.03 | 1.03 | 1.04 | <0.01 |
| PaCO2 (mmHg) with 10 increase | 0.77 | 0.65 | 0.92 | <0.01 |

**Supplemental Table 7. The important variables of SB administration for based on marginal structural cox model for predicting hospital mortality in septic shock patients with acute moderate lacitc acidosis**.

| Variables | Hazard ratio | Lower.95 | Upper.95 | P value |
| --- | --- | --- | --- | --- |
| Use of sodium bicarbonate | 0.44 | 0.20 | 0.97 | <0.05 |
| Hypertension | 0.53 | 0.34 | 0.85 | <0.01 |
| Use of vsopressors | 1.80 | 1.00 | 3.23 | <0.05 |
| Age with 10 years increase | 1.22 | 1.05 | 1.40 | <0.01 |
| Lactate (mmol/L) on admission | 0.87 | 0.81 | 0.92 | <0.01 |
| Lactate solution (L) with 1 increase | 0.86 | 0.77 | 0.96 | <0.01 |
| Lactate (mmol/L) with 0.1 increase | 1.04 | 1.03 | 1.04 | <0.01 |
| PaCO2(mmHg) with 10 increase | 0.65 | 0.53 | 0.81 | <0.01 |

**Supplemental Table 8. Predictors of the use of sodium bicarbonate infusion at each time point during ICU stay in septic patients with acute moderate lactic acidosis**

| Key Characteristics | OR | Lower.95 | Upper.95 | P value |
| --- | --- | --- | --- | --- |
| Demographic information: |  |  |  |  |
| Age with 10 years increase | 1.04 | 0.94 | 1.15 | 0.42 |
| Gender (female as reference) | 0.93 | 0.69 | 1.24 | 0.61 |
| BMI with 5 increase | 0.97 | 0.87 | 1.08 | 0.59 |
| Admission period (before 2014 as reference) | 0.54 | 0.38 | 0.78 | <0.01 |
| Comorbidities (n(%)): |  |  |  |  |
| Hypertension | 0.80 | 0.56 | 1.14 | 0.22 |
| Diabetes | 0.83 | 0.60 | 1.16 | 0.28 |
| Congestive heart failure | 1.21 | 0.86 | 1.70 | 0.28 |
| Chronic pulmonary disease | 0.95 | 0.60 | 1.48 | 0.81 |
| Chronic kidney disease | 0.88 | 0.57 | 1.36 | 0.56 |
| Chronic liver disease | 1.20 | 0.69 | 2.09 | 0.51 |
| The incidence of shock status (n(%)) | | | | |
| Shock | 1.39 | 0.97 | 2.00 | 0.07 |
| Additional respiratory and hemodynamic support, (n(%)): | | | | |
| Mechanical ventilation | 1.14 | 0.70 | 1.87 | 0.59 |
| Vasopressors | 0.73 | 0.47 | 1.12 | 0.15 |
| Laboratory values, (median, (IQR)): | | | | |
| PaCO2(mmHg) with 10 increase | 1.00 | 1.00 | 1.00 | <0.01 |
| pH with 0.1 increase | 0.57 | 0.48 | 0.69 | <0.01 |
| Bicarbonate(mmol/L) with 5 increase | 0.39 | 0.30 | 0.50 | <0.01 |
| Lactate (mmol/L) with 0.1 increase | 1.01 | 1.00 | 1.01 | <0.05 |
| PCO2(mmHg) on admission | 1.02 | 1.00 | 1.04 | <0.05 |
| pH on admission | 1.64 | 0.22 | 12.05 | 0.63 |
| Bicarbonate(mmol/L) on admission | 0.99 | 0.93 | 1.06 | 0.86 |
| Lactate (mmol/L) on admission | 1.00 | 0.95 | 1.07 | 0.89 |
| Lactate solution (L) with 1 increase | 0.91 | 0.83 | 0.99 | <0.05 |

BMI: body mass index

**Supplemental Table 9. Predictors of the use of sodium bicarbonate infusion at each time point during hospital stay in the septic patients with acute moderate lactic acidosis**

| Key Characteristics | OR | Lower.95 | Upper.95 | P value |
| --- | --- | --- | --- | --- |
| Demographic information: | | | | |
| Age with 10 years increase | 1.02 | 0.93 | 1.11 | 0.72 |
| Gender (female as reference) | 0.88 | 0.67 | 1.15 | 0.35 |
| BMI with 5 increase | 1.00 | 0.92 | 1.09 | 0.99 |
| Admission period (before 2014 as reference) | 0.49 | 0.35 | 0.68 | <0.01 |
| Comorbidities (n(%)): |  |  |  |  |
| Hypertension | 0.73 | 0.53 | 1.01 | 0.05 |
| Diabetes | 0.85 | 0.62 | 1.15 | 0.29 |
| Congestive heart failure | 1.19 | 0.87 | 1.63 | 0.26 |
| Chronic pulmonary disease | 0.94 | 0.62 | 1.43 | 0.78 |
| Chronic kidney disease | 0.83 | 0.56 | 1.24 | 0.36 |
| Chronic liver disease | 0.96 | 0.59 | 1.58 | 0.88 |
| The incidence of shock status (n(%)) | | | | |
| Shock | 1.21 | 0.87 | 1.68 | 0.26 |
| Additional respiratory and hemodynamic support, (n(%)): | | | | |
| Mechanical ventilation | 1.08 | 0.68 | 1.69 | 0.75 |
| Vasopressors | 0.84 | 0.58 | 1.22 | 0.36 |
| Laboratory values, (median, (IQR)): | | | | |
| PCO2(mmHg) with 10 increase | 0.74 | 0.63 | 0.86 | <0.01 |
| pH with 0.1 increase | 0.56 | 0.46 | 0.68 | <0.01 |
| Bicarbonate(mmol/L) with 5 increase | 0.36 | 0.28 | 0.45 | <0.01 |
| Lactate (mmol/L) with 0.1 increase | 1.01 | 1.00 | 1.01 | <0.05 |
| PCO2(mmHg) on admission | 1.03 | 1.01 | 1.05 | <0.01 |
| pH on admission | 1.03 | 0.15 | 7.11 | 0.97 |
| Bicarbonate(mmol/L) on admission | 1.03 | 0.97 | 1.09 | 0.29 |
| Lactate (mmol/L) on admission | 1.01 | 0.96 | 1.07 | 0.63 |
| Lactate solution (L) with 1 increase | 0.90 | 0.84 | 0.97 | <0.01 |

BMI: body mass index

**Supplemental Table 10. Predictors of the use of sodium bicarbonate infusion at each time point during hospital stay in the septic shock patients with acute moderate lactic acidosis**

| Key Characteristics | OR | Lower.95 | Upper.95 | P value |
| --- | --- | --- | --- | --- |
| Demographic information: | | | | |
| Age with 10 years increase | 1.06 | 0.96 | 1.18 | 0.27 |
| Gender (female as reference) | 1.04 | 0.75 | 1.43 | 0.81 |
| BMI with 5 increase | 1.03 | 0.92 | 1.16 | 0.58 |
| Admission period (before 2014 as reference) | 0.48 | 0.33 | 0.71 | <0.01 |
| Comorbidities (n(%)): |  |  |  |  |
| Hypertension | 0.66 | 0.46 | 0.96 | <0.05 |
| Diabetes | 0.88 | 0.61 | 1.27 | 0.49 |
| Congestive heart failure | 1.16 | 0.80 | 1.68 | 0.42 |
| Chronic pulmonary disease | 0.91 | 0.54 | 1.54 | 0.74 |
| Chronic kidney disease | 0.55 | 0.31 | 0.95 | <0.05 |
| Chronic liver disease | 0.93 | 0.52 | 1.66 | 0.79 |
| Additional respiratory and hemodynamic support, (n(%)): | | | | |
| Mechanical ventilation | 0.93 | 0.54 | 1.59 | 0.79 |
| Vasopressors | 0.83 | 0.50 | 1.38 | 0.48 |
| Laboratory values, (median, (IQR)): | | | | |
| PCO2(mmHg) with 10 increase | 0.75 | 0.63 | 0.88 | <0.01 |
| pH with 0.1 increase | 0.54 | 0.43 | 0.68 | <0.01 |
| Bicarbonate(mmol/L) with 5 increase | 0.38 | 0.29 | 0.50 | <0.01 |
| Lactate (mmol/L) with 0.1 increase | 1.01 | 1.00 | 1.02 | <0.01 |
| PCO2(mmHg) on admission | 1.03 | 1.01 | 1.05 | <0.01 |
| pH on admission | 0.98 | 0.12 | 8.38 | 0.99 |
| Bicarbonate(mmol/L) on admission | 1.05 | 0.98 | 1.12 | 0.18 |
| Lactate (mmol/L) on admission | 1.00 | 0.93 | 1.06 | 0.93 |
| Lactate solution (L) with 1 increase | 0.89 | 0.81 | 0.96 | <0.01 |

BMI: body mass index
